# MicroRNAs Are Involved in the Development of Morphine-Induced Analgesic Tolerance and Regulate Functionally Relevant Changes in *Serpini1*

**DOI:** 10.3389/fnmol.2016.00020

**Published:** 2016-03-24

**Authors:** Jenica D. Tapocik, Kristin Ceniccola, Cheryl L. Mayo, Melanie L. Schwandt, Matthew Solomon, Bi-Dar Wang, Truong V. Luu, Jacqueline Olender, Thomas Harrigan, Thomas M. Maynard, Greg I. Elmer, Norman H. Lee

**Affiliations:** ^1^National Institute of Alcohol Abuse and Alcoholism, National Institutes of HealthBethesda, MD, USA; ^2^Department of Pharmacology and Physiology, The George Washington UniversityWashington, DC, USA; ^3^Department of Psychiatry, Maryland Psychiatric Research Center, University of Maryland School of MedicineBaltimore, MD, USA

**Keywords:** miR-27a, miR-9, Dicer, opioid, plasticity

## Abstract

Long-term opioid treatment results in reduced therapeutic efficacy and in turn leads to an increase in the dose required to produce equivalent pain relief and alleviate break-through or insurmountable pain. Altered gene expression is a likely means for inducing long-term neuroadaptations responsible for tolerance. Studies conducted by our laboratory (Tapocik et al., 2009) revealed a network of gene expression changes occurring in canonical pathways involved in neuroplasticity, and uncovered miRNA processing as a potential mechanism. In particular, the mRNA coding the protein responsible for processing miRNAs, *Dicer1*, was positively correlated with the development of analgesic tolerance. The purpose of the present study was to test the hypothesis that miRNAs play a significant role in the development of analgesic tolerance as measured by thermal nociception. *Dicer1* knockdown, miRNA profiling, bioinformatics, and confirmation of high value targets were used to test the proposition. Regionally targeted *Dicer1* knockdown (via shRNA) had the anticipated consequence of eliminating the development of tolerance in C57BL/6J (B6) mice, thus supporting the involvement of miRNAs in the development of tolerance. MiRNA expression profiling identified a core set of chronic morphine-regulated miRNAs (miR's 27a, 9, 483, 505, 146b, 202). Bioinformatics approaches were implemented to identify and prioritize their predicted target mRNAs. We focused our attention on miR27a and its predicted target serpin peptidase inhibitor clade I (*Serpini1*) mRNA, a transcript known to be intricately involved in dendritic spine density regulation in a manner consistent with chronic morphine's consequences and previously found to be correlated with the development of analgesic tolerance. *In vitro* reporter assay confirmed the targeting of the *Serpini1* 3′-untranslated region by miR27a. Interestingly miR27a was found to *positively* regulate *Serpini1* mRNA and protein levels in multiple neuronal cell lines. Lastly, Serpini1 knockout mice developed analgesic tolerance at a slower rate than wild-type mice thus confirming a role for the protein in analgesic tolerance. Overall, these results provide evidence to support a specific role for miR27a and *Serpini1* in the behavioral response to chronic opioid administration (COA) and suggest that miRNA expression and mRNA targeting may underlie the neuroadaptations that mediate tolerance to the analgesic effects of morphine.

## Introduction

Opioid analgesics are the foundational treatment in pain management. No other drug provides better relief from the sensory and affective components of pain. Unfortunately, long-term opioid treatment results in reduced therapeutic efficacy and in turn leads to an increase in the dose required to produce equivalent pain relief, break-through, or insurmountable pain (Carroll et al., 2004; Angst and Clark, 2006; Colameco et al., 2009; Fishbain et al., 2009; Silverman, 2009; Zernikow et al., 2009; Bekhit, 2010; Crofford, 2010), increased adverse side-effects (Mercadante, 1999; Mercadante and Portenoy, 2001) and may progress to opioid dependence and addiction (Banta-Green et al., 2009; Shurman et al., 2010). Despite the clinical importance of diminishing the development of analgesic tolerance and decades of research into the neurobiological consequences of chronic opioid administration (COA), the mechanism is still poorly understood and only limited intervention strategies are available (Huxtable et al., 2011; Labianca et al., 2012).

The subjective experience of pain and its alleviation by opioids involve direct sensory perception and cognitive and affective response (Price, 2000; Rainville, 2002). The direct, affective and cognitive/associative components of pain and analgesia are mediated throughout central nervous system (CNS) regions containing μ-opiate receptors including periaqueductal gray, amygdala, lateral habenula, dorsal raphe, anterior cingulate, and prefrontal cortex (PFC) (Pastoriza et al., 1996; Wagner et al., 2001; Craggs et al., 2007; Wager et al., 2007; Petrovic et al., 2010; Stockton and Devi, 2014; Zeidan et al., 2015). A clear consensus on specific canonical pathways altered by COA has not been reached, however complex intracellular neuroadaptations in direct response to opioid administration (Chu et al., 2008; Drdla et al., 2009; Ueda and Ueda, 2009; Abul-Husn et al., 2011; Ziolkowska et al., 2012; Williams et al., 2013) initiate complex synaptic reorganization characterized by altered neurotransmission (Abul-Husn et al., 2011) and synaptic architecture (Russo et al., 2010; Abul-Husn et al., 2011; Stockton and Devi, 2014) such as decreased spine density (Robinson et al., 2002), cytoskeletal structure (Hou et al., 2009; Li et al., 2009), cell size (Russo et al., 2007), and neurotransmitter relevant protein distribution (Xu et al., 2003; Mickiewicz and Napier, 2011). Neuroadaptations initiated by COA are additionally contingent upon context-dependent associative components (Tiffany et al., 1991; Cox and Tiffany, 1997; Mitchell et al., 2000; Miguez et al., 2014) that together underlie the behavioral effects of repeated opioid administration. At a genomics level, recent studies in our laboratories anchored morphine-induced region-specific mRNA expression patterns to specific behavioral endpoints such as analgesic tolerance (Tapocik et al., 2009) and morphine self-administration (Tapocik et al., 2013) in order to improve the likelihood of identifying causal genes (Letwin et al., 2006; Reiner-Benaim et al., 2007; Tapocik et al., 2009). Bioinformatics analysis of these data sets revealed a statistical over-representation of gene expression changes occurring in canonical pathways responsible for regulating neuroplasticity and the processing of miRNAs.

Given the significant amount of support for neuroplasticity changes following COA (Kauer and Malenka, 2007; Kalivas and O'Brien, 2008; McClung and Nestler, 2008; Stockton and Devi, 2014), altered miRNA expression is a promising candidate to coordinate the complex response. MiRNAs are a class of noncoding RNAs that regulate gene expression by targeting mRNAs for translation inhibition and/or mRNA degradation (He and Hannon, 2004). These molecules are thought to regulate as much as one-third of the transcribed genome (Stefani and Slack, 2008) and can mediate short and long term neuroplasticity including structural regulation by sculpting dendritic architecture (Kosik, 2006; Schratt et al., 2006; Wayman et al., 2008). A recent series of promising studies specifically confirmed a role for the miRNA let-7 in the development of analgesic tolerance following high opioid doses (He et al., 2010; He and Wang, 2012) and several other miRNAs following opioid administration (miR-103/miR-107, -124, 190, -23b, -133b, -339) (Wu et al., 2009, 2013; Sanchez-Simon et al., 2010; Zheng et al., 2010a,c; Ni et al., 2013; Gonzalez-Nunez et al., 2014; Lu et al., 2014; Qiu et al., 2015). Despite the strategic capacity of miRNAs to mediate dynamic and long-term neuroplasticity, surprisingly few studies have determined their *global* differential regulation following administration of chronic opioids (Wu et al., 2008, 2009, 2013; Dave and Khalili, 2010; Zheng et al., 2010c; Gonzalez-Nunez et al., 2014; Tang et al., 2014).

Based upon our discovery that *Dicer1*, the rate limiting enzyme in miRNA production (Kosik and Krichevsky, 2005), was positively associated with the development of tolerance (Tapocik et al., 2009) and the suggested miRNA involvement in morphine self-administration (Tapocik et al., 2013), we hypothesized that COA alters miRNA expression profiles in a manner that modulates relevant neuroplasticity mRNA expression. Several experimental conditions and genotypes were exposed to repeated administration of therapeutically relevant morphine doses to assess the altered tolerance development in a thermal nociception assay. ShRNA-mediated *Dicer1* knockdown and genotypes that are sensitive and resistant to the development of tolerance (C57BL/6J and DBA/2J, respectively) were utilized to provide evidence to support a significant role for miRNA processing in analgesic tolerance. Regional miRNA expression profiling following COA in the C57BL/6J mice and refinement by comparison to tolerance-insensitive DBA/2J mice led to the identification of intriguing synaptic organization miRNA candidates and the identification of a unique miRNA-mRNA-protein axis putatively involved in synaptic organization.

## Materials and methods

### Animals

Adult male C57BL/6J (B6), DBA/2J (D2), and B6.129-Serpini1tm1Dpw/J (Serpini1 KO) mice (Jackson Laboratories, Bar Harbor, ME), 60–120 days old and weighing ~21–28 g at the start of the experiment were used. Only males were tested in order to permit miRNA-mRNA pairing analysis by combining our previous mRNA data set obtained in males (Tapocik et al., 2009) with our current miRNA analysis (see below). All animals were experimentally naive, housed in a temperature-controlled room (21°C; 0700–1900 lights on), and given free access to chow and tap water during the entire experimental procedure. The treatment of animals followed the Principles of Laboratory Animal Care (NIH publication no.86-23, 1996) and was approved by the Institutional Animal Care and Use Committee of the University of Maryland School of Medicine.

### Analgesic tolerance assessment (general description)

The ability of a high, therapeutically relevant dose of morphine to maintain analgesic efficacy across three spaced administrations (48 h) was used as the endpoint for tolerance. The specific morphine dose for each condition and genotype tested are given in each respective section. Saline vehicle controls accounted for any potential group differences across repeated exposure to the hot-plate testing and were utilized in the calculation for percent maximal possible effect (%MPE) (see analysis below). Morphine-induced analgesic effects were determined on a hot-plate maintained at a constant temperature of 55°C. The subject was placed on the hot- plate 15 min post sub-cutaneous (s.c.) saline or morphine (Sigma-Aldrich, St Louis, MO) injection (0.01 ml/kg). A large 25 cm diameter circular plastic container served to restrict mice to the hot-plate. Saline or the ED90 dose of morphine was administered between the hours of 8:00–11:00 a.m. The dependent measure used to assess thermal antinociception was the latency to paw lick (hind or front). A cut-off time of four times each genotype's saline control value was used to avoid tissue damage and to factor in important genotype-dependent differences in baseline nociception (Elmer et al., 1998). The analgesic tests are described in the order they were conducted.

Data were analyzed as the %MPE. The saline vehicle group for each respective genotype was used as the baseline latency in the following formula:

$$100^{*}\left[\frac{\left(\left(\text{test latency}\right)-\left(\text{saline baseline latency}\right)\right)}{\left(\left(4^{*}\text{baselinelatency}\right)-\left(\text{baselinelatency}\right)\right)}\right]$$

Analgesic experiments were analyzed using two-way repeated measures ANOVA (genotype × injection number) and co-varied for treatment order within a cage.

### Lentiviral shRNA dicer

Four experimental groups of B6 mice were used in this experiment: *Nonsense* shRNA-morphine, *Nonsense* shRNA-saline, *Dicer1* shRNA-morphine, *Dicer1* shRNA-saline (*n* = 6, 7, 6, and 6, respectively). Four weeks following *nonsense* shRNA or *Dicer1* shRNA injection into the PFC (see full virus preparation and surgery description below), mice were administered 31.2 mg/kg morphine (s.c.) or saline (s.c.) once every other day over a 5-day period (total 3 treatments) and assessed for tolerance by the hot plate test following the last treatment. The morphine dose represents the ED_90_ in B6 mice (see below). Saline or the morphine was administered s.c. between the hours of 8:00–11:00 a.m. Brains were harvested for immunohistochemistry confirmation following the last treatment.

### B6 and D2 mice

Analgesic tolerance was assessed in four groups of mice; B6 saline control, B6 morphine, D2 saline control, D2 morphine (Total *N* = 10, 10, 9, and 8, respectively). Following assessment of analgesic tolerance, brain tissue was harvested for miRNA expression profiling (B6; *n* = 4 each for saline and morphine treatment) or rtPCR confirmation of candidate miRNAs (B6; *n* = 6 each for saline and morphine treatment) and determination of candidate miRNA expression levels in tolerant-resistant mice (D2; *n* = 4 each for saline and morphine treatment). As stated previously, saline controls accounted for any potential differences across repeated exposure to the hot-plate testing and were utilized in the calculation for percent maximal possible effect (%MPE) (see analysis below). The dose of morphine required to produce ~90% of the %MPE for B6 and D2 was used to induce tolerance since tolerance to the analgesic effects of morphine develops at a rate directly related to the magnitude of the initial pharmacological effect (Cox and Tiffany, 1997). If the same dose of morphine was used to induce tolerance in subjects that differ significantly in the acute analgesic potency of morphine, tolerance could appear to develop more rapidly in sensitive genotypes given the greater magnitude of the initial pharmacological effect. This confound would affect the tolerance outcome and interpretation. Thus, the experiment accounts for this by using genotype-specific doses within a “therapeutic” range, (ED_90_). The ED_90_ dose of morphine for the B6 and D2 mice was 31.2 and 7.7 mg/kg, respectively, as determined by full dose-effect curve analysis in a previous experiment (see Figure 1 of Tapocik et al., 2009). Mice were administered 31.2 mg/kg morphine (s.c.) or saline (s.c.) once every other day over a 5-day period (total 3 treatments) and assessed for tolerance by the hot plate test after each treatment.

### *Serpini1* KO

Four groups of mice were tested in this experiment; *Serpini1* KO saline control, *Serpini1* KO morphine, B6 saline control, B6 morphine (*n* = 8, 8, 6, and 6, respectively) as described above. For the experiments using *Serpini1* KO mice the B6 mouse served as the WT control (per Jackson Laboratory recommendation). While the ED_90_ dose for B6 mice is 31.2 mg/kg (see above), a slightly higher dose (42.0 mg/kg) was used for these resource intensive KO and B6 controls in order to ensure full tolerance following three drug administrations.

### Lentiviral delivery of shRNA targeting *Dicer1*

We had previously demonstrated that *Dicer1* expression in the PFC was positively correlated with the development of analgesic tolerance across a panel of inbred mouse strains (Tapocik et al., 2009). B6 mice, a strain exhibiting the highest levels of *Dicer1* mRNA, displayed the greatest tolerance following COA. Conversely, the D2 strain had the lowest levels of *Dicer1* and exhibited the least tolerance. Consequently, the effects of *Dicer1* knockdown on analgesic tolerance were investigated.

Lentiviral production and shRNA vector construction were performed as previously described (Riz et al., 2010) with the following modifications. shRNA design was accomplished using a selection program available at http://jura.wi.mit.edu/bioc/siRNAext/. *Dicer1* shRNA targeting sequence 5′CGC CGATCTCTAATTACGTAATAGTGAAGCCACAGATGTA TTACGTAATTAGAGATCG GCG-3′ or nonsense sequence 5′CCA AATTATACCTAC ATTGCTTAGTGAAGCCACAGATGTAAGCAATGTAGGTATAAT TTGG-3′ was cloned into the shuttle vector pENTR for recombination with the pLenti6.2 destination vector containing green fluorescent protein (GFP) (Gateway Technology, Life Technologies, Grand Island, NY). A BLAST search on shRNA sequences was performed against the mouse RefSeq database to ensure target specificity. The destination plasmid pLenti6.2 with shRNA targeting sequence, VSV-G protein envelope plasmid pMD2.G, and packaging plasmid pSPAX2 (kindly provided by J. Silvio Gutkind) were transiently transfected into human embryonic kidney 293FT cells (Life Technologies) using ExGen 500 (Fermentas Inc., Glen Bernie, MD), and the supernatant containing vesicular stomatitis virus (VSV)-G glycoprotein-pseudotyped lentiviral vector particles was collected and purified. Lentiviral vector particles were titered by FACS and re-suspended at a concentration ~2 million transducing viral particles per 0.5 microliter.

Knockdown efficiency of shRNA was tested by Lipofectamine 2000 (Life Technologies Corp., Grand Island, NY)-mediated transfection of mouse neuronal cell line N1E-115 cells with the *Dicer1* targeting or nonsense sequence cloned into pSuper (Addgene, Cambridge, MA) for 24 h. Total RNA was isolated from transfected cells using the RNAeasy purification kit (Qiagen Inc., Valencia, CA), and Real-time quantitative reverse transcription (qRT)-PCR was performed using the TaqMan Universal PCR Master Mix (Applied Biosystems, Foster City, CA) and *Dicer1*-specific primers 5′CTTGGATTCCTGGTG GAAGA-3′ (forward) and 5′GAGCCCTGA GAATGGATGAA-3′ (reverse) and cyclophilin-like 4 control gene primers 5′TGTTTCCTCATCAGC ACAGG-3′ (forward) and 5′AGGTCAGTG CCGAGTGTCTT-3′ (reverse). Real-time qRT-PCR parameters were as follows: denaturing at 95°C for 15 min and annealing/extension at 60°C for 1 min for a total of 30 cycles performed in the 7300 Real-time PCR System (Applied Biosystems, Foster City, CA). Normalized expression was calculated using the comparative Ct method and fold change was derived from the equation 2^−ΔΔCt^.

### Surgery, lentiviral particle injection, and tolerance assessment

Lentiviral vector particles containing shRNA targeting *Dicer1* or nonsense sequence (control) was injected bilaterally into the PFC of B6 mice. The stereotaxic coordinates were as follows: anterior-posterior +1.70 mm, medial-lateral ± 0.6 mm, dorsal-ventral −1.75 mm (relative to Bregma). Packaged viral particles (~2 million transducing units) were injected at a volume of 0.5 microliters per hemisphere at a rate of 0.25 μl/min (2 min/injection/hemisphere, 0.26 mm needle diameter, Plastics One, Roanoke VA). The needle was left in for an additional 2 min to allow the solution to diffuse. Mice were treated with rimadyl (5.0 mg/kg, i.p.) once per day to provide analgesia for 2 days.

Four weeks following shRNA injection, the mice were treated with an ED_90_ dose of morphine (31.2 mg/kg) or saline once every other day over a 5-day period (total 3 treatments) and assessed for tolerance by the hot plate test following the last treatment as described in detail above. Four experimental groups of B6 mice were used: *Dicer1* shRNA-morphine (ED_90_); *Dicer1* shRNA-saline; *Nonsense* shRNA-morphine (ED_90_); *Nonsense* shRNA-saline. Brains were harvested for immunohistochemistry confirmation.

### Immunohistochemistry

Lentiviral-mediated shRNA placement and expression was analyzed by immunohistochemistry with anti-GFP and anti-Dicer1 antibodies. Each mouse was anesthetized with ketamine- (100 mg/kg, i.p.) and xylazine- (16 mg/kg, i.p.), 10 min later perfused with 4% paraformaldehyde, and brains were extracted. The brains were immersed in formalin for 24 h and then switched to 18% sucrose solution for 48 h. The brains were snap frozen in dry ice and stored in a −80°C freezer until further use. The formalin fixed brains mounted in OTC embedding compound (Thermo Fisher Scientific, Waltham, MA) were sectioned on a −20°C cryostat (Leica Microsystems Inc., Wetzlar, Germany) at 20 μm intervals and mounted serially on poly-L-lysine-coated slides and stored in −80°C until labeling. The slices were rinsed with 1X PBS three times for 5 min, rinsed two times for 5 min in 0.1% glycine (dissolved in 1X PBS), followed by a 1 h incubation in 50 mM ammonium chloride (NH_4_Cl dissolved in 1X PBS). Slices were rinsed again three times for 5 min with 1X PBS, blocked in the blocking buffer for 1 h (3% serum, 0.3% triton X, dissolved in 1X PBS), and then incubated with the primary antibodies overnight at 4°C (1% serum, 0.3% triton X, dissolved in 1X PBS). The primary antibodies were anti-GFP (chicken anti-GFP, #A10262; 1:1000 dilution; Invitrogen, Carlsbad, CA; A11122) and anti-Dicer1 (rabbit anti-Dicer1, #71691; 1:250 dilution; Novus Littleton, CO). Slices were then rinsed three times for 5 min with 1X PBS and were treated with the appropriate secondary antibodies for 2 h (1% serum, 0.3% triton X, dissolved in 1X PBS): at 1:200 dilution with goat anti-rabbit red Alexafluor-594 and goat anti-chicken green Alexafluor-488 (Molecular Probes, Eugene Oregon). Slices were rinsed one time for 5 min with 1X PBS and then stained with DAPI for 30 min (300 nM: 1 μl DAP1 into 1 ml H_2_O; above solution in 49 ml of 1X PBS). DAPI counterstain was utilized to determine the cytoarchitecture of anatomical regions. DAPI was washed off by rinsing three times for 5 min in 1X PBS and one time in double distilled H_2_O. Slides were coverslipped and images were captured on an Olympus (Center Valley, PA) fluorescence microscope and an Olympus FluoView 500 confocal microscope (Center Valley, PA). The positive fluorescence cells were mapped within the PFC at the location of the injection site using the mouse brain stereotaxic coordinates (Paxinos and Franklin, 2001). The GFP-positive cells were mapped within the PFC at the location of the injection site using the mouse brain stereotaxic coordinates (Paxinos and Franklin, 2001), and fluorescence pixel values for Dicer1 within GFP-positive cells were quantified at 20X magnification using Photoshop 12.0 software.

### miRNA expression profiling

Brain tissue was harvested in a subset of the B6 mice 3 h after the end of tolerance testing on the last test day (see analgesia testing above; *n* = 4 each for saline and morphine treated mice). Each mouse was euthanized by CO_2_ asphyxiation. A Plexiglas brain mold (David Kopf Instruments, Tujunga, CA) was used to slice the fresh whole brain into an appropriate coronal slice. Using the atlas of Paxinos and Franklin (2001) as a reference, the PFC was taken in the following parameters: interaurally 5.90–4.90 mm, from the dorsal-most point to 2.25 mm ventrally at a medial-lateral width of 1 mm centered on midline. The PFC dissection includes the prelimbic, infralimbic, cingulate and portions of the secondary motor cortex. MiRNA isolation from PFC, miRNA quality control, labeling and hybridization onto Agilent SurePrint Mouse miRNA microarrays, and data extraction, normalization and statistical analyses were performed as previously described (Wang et al., 2010). Statistical differences in miRNA expression between saline and morphine administration were analyzed by an ANOVA with 10% false discovery rate (FDR) for multiple test correction on the ArrayStat software package (Amersham Biosciences Corp.). MiRNA data has been deposited to the Gene Expression Omnibus (GEO) and can be assessed using Accession number GSE75893.

### Real-time qRT-PCR validation

Validation of miRNA expression array results was accomplished by qRT-PCR of total RNA isolated from separate B6 and D2 animals (see analgesia testing above; *n* = 6, 6, 4, and 4 for B6 saline, B6 morphine, D2 saline, and D2 morphine, respectively). Tolerance was assessed as described above. Testing and PFC harvest was conducted as described above. Isolation of total RNA from PFC and qRT-PCR was performed using the NCode EXPRESS SYBR GreenER microRNA qRT-PCR Kit (Invitrogen, Carlsbad, CA), as previously described (Wang et al., 2010). Briefly, poly(A)-tailed reverse transcription (RT) reaction products were mixed with Express SYBR green qPCR SuperMix, miRNA-specific forward primer (Supplementary Table 1) and universal qPCR primer and subjected to 40 cycles of 95°C for 15 s, and 60°C for 1 min performed in a 7300 Real-time PCR System (Applied Biosystems, Foster City, CA). U6 snRNA was used as the internal standard reference in the qRT-PCR reactions. Normalized expression was calculated using the comparative Ct method and fold change was derived from the equation 2^−ΔΔCt^ for each miRNA.

### Integrative analysis of miRNA and mRNA microarray data

Target mRNA predictions were determined for the differentially expressed miRNAs, and the number of target mRNAs were cross-referenced with 78 differentially regulated mRNAs determined in the earlier expression profiling analyses (Tapocik et al., 2009). The mRNA data from Tapocik et al. (2009) can be assessed at the *Journal of Neuroscience* website at http://www.jneurosci.org/. MiRNA target analysis was conducted using the microRNA.org website (Betel et al., 2008). “Good” miRSVR and PhastCon scores with cutoffs based on algorithms in microRNA.org were used to determine predicted miRNA-mRNA interactions (Betel et al., 2010). The scores determined proper association of miRNA expression patterns with our experimentally observed mRNA expression results. Follow-up targets for confirmation were prioritized based on information provided by miRNA-mRNA expression pairing, hypergeometric testing (see below), DAVID Gene Ontology, gene network and canonical analysis (Ingenuity Pathway Analysis, Redwood City, CA).

### Hypergeometric testing

To determine whether any of the differentially expressed miRNAs “over-targeted” genes that were differentially regulated, we conducted an over-representation analysis of targeting events, similar to that used by Lewohl et al. (2011). Hypergeometric tests were conducted for each differentially expressed miRNA. Correction for multiple testing was carried out using the linear step-up method for a 10% FDR (Benjamini and Hochberg, 1995).

### miR-3′UTR binding luciferase assay

N2A cells were grown on poly-D-lysine in 96-well format to a 70–85% confluency and transfected using Lipofectamine 2000 (Life Technologies Corp., Grand Island, NY) with 200 ng of psiCHECK2-*Serpini1*-3′ UTR plasmid and 5 pmol of *Mus musculus* (mmu)-miR-27a, mmu-miR-9, or nonsense mimics (Exiqon, Woburn, MA). At 48 h post-transfection, cells were lysed and luciferase reporter activity was assayed using the Dual-Luciferase Reporter Assay System (Promega, Madison, WI). The activities of *firefly* and *Renilla* luciferases were measured sequentially from a single sample in a FLUORstar-BMG luminometer (IMGEN, Alexandria, VA) for 60 s each immediately after addition of Luciferase Assay Reagent II for *firefly* luciferase or Stop and Glo Reagent for *Renilla* luciferase. *Renilla* luciferase values were normalized to control *firefly* luciferase levels (transcribed from the same vector but not affected by 3′ UTR tested) and averaged across 3–5 well repetition, resulting in 3–5 technical and 3 biological replicates for each condition (miR-9, 27-, 206, and nonsense control).

### MiR-27a regulation of serpini1 protein levels in mouse neuronal cell lines

Computational prediction that miR-27a targets *Serpini1* was verified by individually transfecting mouse neuronal cell lines N2A and N1E115 with 5 pmol of mmu-miR-27a or nonsense control mimic (Exiqon, Woburn, MA). At 48 h post-transfection, lysates were isolated from cell lines for western blot analysis of serpini1 (see below). Four and eight independent experiments were performed in N1E-115 and N2A cells, respectively. Each independent experiment was defined as a culture of cells treated with NS control mimic and a separate culture treated with the miR-27a mimic.

### Western blot analysis

Cell lysates from PFC of B6 mice or neuroblastoma cell lines (N2A and N1E115) were subjected to western blot analysis as previously described (Wang et al., 2010). Briefly, protein concentration of lysates was determined using NanoOrange Protein Quantitation Kit (Molecular Probes, Invitrogen, Eugene, OR). Twenty-five micrograms of protein lysate from cell lines or 50 μg from PFC was separated using standard SDS-PAGE with precast 4–20% gels (BioRad, Hercules, CA) and transferred to PVDF membranes using standard transfer conditions. We employ two approaches in order to ensure equal loading of total protein lysate in each lane of the western blot. First, we stain the blots with ponceau to ensure overall equal loading of protein lysates. Second, we normalize our calculations with beta-actin. Next, membranes were washed and blocked for at least 1 h using BSA or powdered milk, followed by sequential incubations of primary antibody against Serpini1 and secondary antibody conjugated to horseradish peroxidase (HRP). Afterwards, membranes were washed and incubated with enhanced chemiluminescence substrate (Perkin Elmer, Waltham, MA) for autoradiography. Films were scanned using a Personal Densitometer SI (Molecular Dynamics, Sunnyvale, CA) and protein bands quantified using ImageQuant software. Membranes were stripped and then incubated with β-actin antibody for quantification and normalization. All antibodies used in this study are mouse monoclonal anti-Serpini1 and anti-β-actin (Sigma-Aldrich, Saint Louis, MO), rabbit polyclonal anti-Dicer1 (Novus Biologics, Littleton, CO), and goat anti-mouse and anti-rabbit secondary antibody conjugated to HRP (Santa Cruz Biotechnology, Santa Cruz, CA).

## Results

### Causal role of *Dicer1* in response to chronic morphine

**Figure 1:**
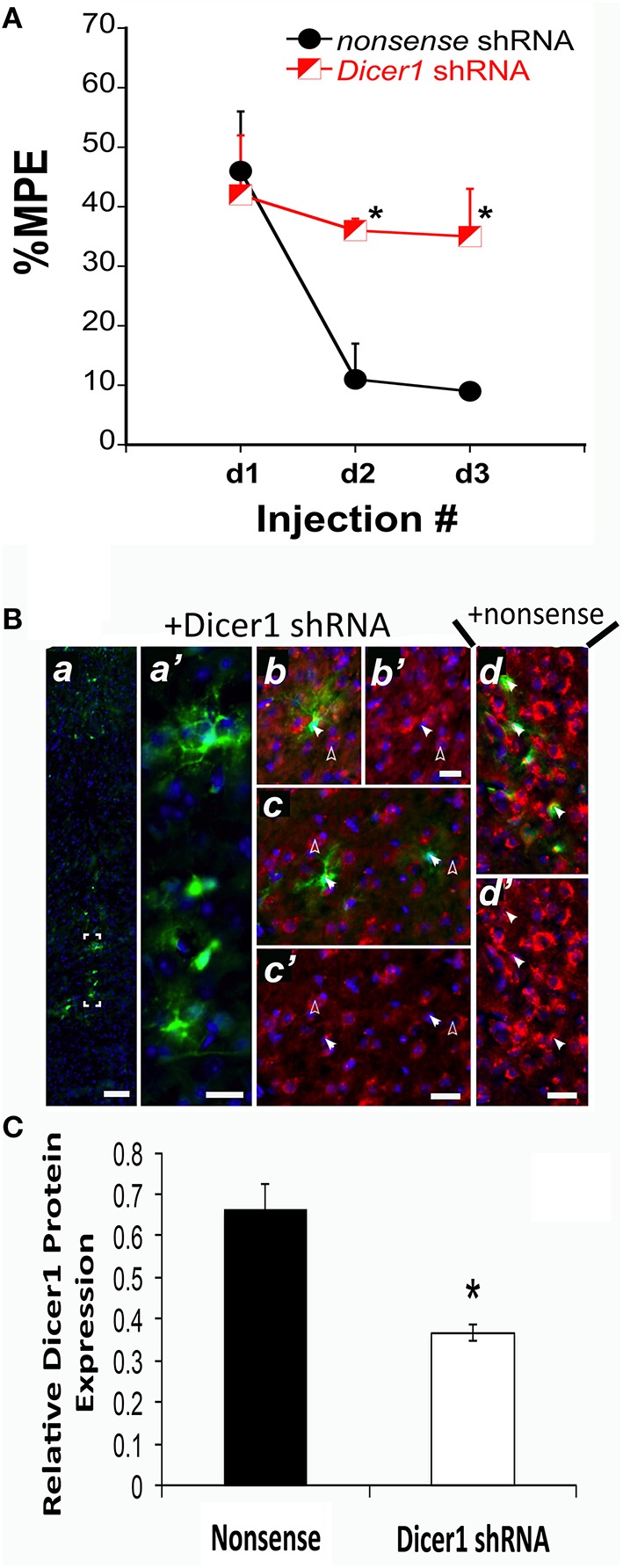
*****s***hRNA targeting ***Dicer1*** in B6 mice significantly reduced the development of tolerance. (A)** The %MPE following 31.2 mg/kg morphine (s.c.) across three injections each spaced 48 h apart significantly decreased in nonsense control mice and not in Dicer1 shRNA treated mice. ^*^Significantly different than nonsense control (*P* < 0.030, 0.001); (*n* = 6,5 for Dicer1 shRNA and nonsense controls, respectively). **(B)**, Immunohistochemistry was performed on behaviorally phenotyped animals in **(A)**. GFP-positive PFC cells indicate successful transduction with lentivirus carrying *Dicer* shRNA. GFP-staining was observed along the injection track in the PFC **(a)**. Bracketed region in A shown enlarged **(a**′**)**. To examine the efficacy of the shRNA-mediated *Dicer1* knockdown in GFP-positive PFC cells, Dicer protein was co-stained by immunofluorescence with a Dicer1-specific antibody (red). Nuclear DNA was co-stained with DAPI (blue channel). In GFP-positive PFC cells containing *Dicer1* shRNA (solid arrows in **b,c**), perinuclear Dicer1 expression is significantly reduced (solid arrows in **b**′**,c**′). By comparison, untransduced neighboring cells not expressing GFP (open arrows in **b,c**) have high expression of Dicer1 (open arrows in **b**′**,c**′). In cells transduced with lentivirus delivering nonsense shRNA control, GFP-positive cells (closed arrows in **d**) retained strong perinuclear Dicer1 protein expression (closed arrows in **d**′). Immunofluorescent images are representative from six animals transduced with lentivirus carrying *Dicer1* shRNA and chronically treated with morphine, and five animals transduced with lentivirus carrying *nonsense* shRNA control and chronically treated with morphine. Scale bars (inset white lines) in **(a)** = 100 μm and **(b–d)** = 25 μm. **(C)** Quantification of Dicer1 immunofluorescence in the PFC **(B)**. Bar graph represents relative Dicer1 protein levels in GFP-expressing cells from control (nonsense shRNA) and Dicer1 shRNA-treated animals. A total of 31 and 40 well-demarcated GFP-expressing cells from control and Dicer1 knockdown animals, respectively were quantified as described in Materials and Methods Section. ^*^Significantly different from nonsense shRNA-treated animals (*P* < 0.0001).

We previously established that *Dicer1* mRNA expression was negatively correlated with the %MPE of morphine after COA (Tapocik et al., 2009). Therefore, we conducted *in vivo* studies to identify the causal role of Dicer1 in response to chronic morphine. Prior to initiating the *in vivo* studies, the effectiveness of an EmGFP-*Dicer1* shRNA cassette to knockdown *Dicer1* mRNA was tested by transducing mouse N1E-115 neuroblastoma cells. Transfection resulted in an 82 ± 4.5% knockdown compared to nonsense control as determined by qRT-PCR (*t* = 4.0, df-6 *P* = 0.007). *In vivo*, disrupting *Dicer1* expression via shRNA in the PFC of B6 mice did not alter baseline sensitivity to thermal stimuli; vehicle baseline latencies were 6.4, 7.6, and 7.7 s for nonsense vehicle controls and 6.4, 9.4, and 7.4 s for *Dicer1* shRNA controls across injection days 1, 2, and 3, respectively. However, *Dicer1* shRNA treated mice failed to develop tolerance compared to the nonsense treated control mice [*F*_(*Virus*)_ *df*_(1, 9)_ = 7.31; *p* = 0.0269] (Figure 1A). In each of the *Dicer1* shRNA injected brains, there was diminished expression of perinuclear Dicer1 protein in the GFP-positive cells (Figure 1B). In contrast, animals injected in the PFC with nonsense shRNA exhibited strong expression of perinuclear Dicer1 protein in the GFP-positive cells (Figure 1B). Quantified fluorescence pixel values for Dicer1 within GFP-positive cells revealed a significant reduction following Dicer1 shRNA-treatment (*t* = 5.20, *p* < 0.0001) (Figure 1C).

### Changes in miRNA expression are associated with tolerance susceptibility

**Figure 2:**
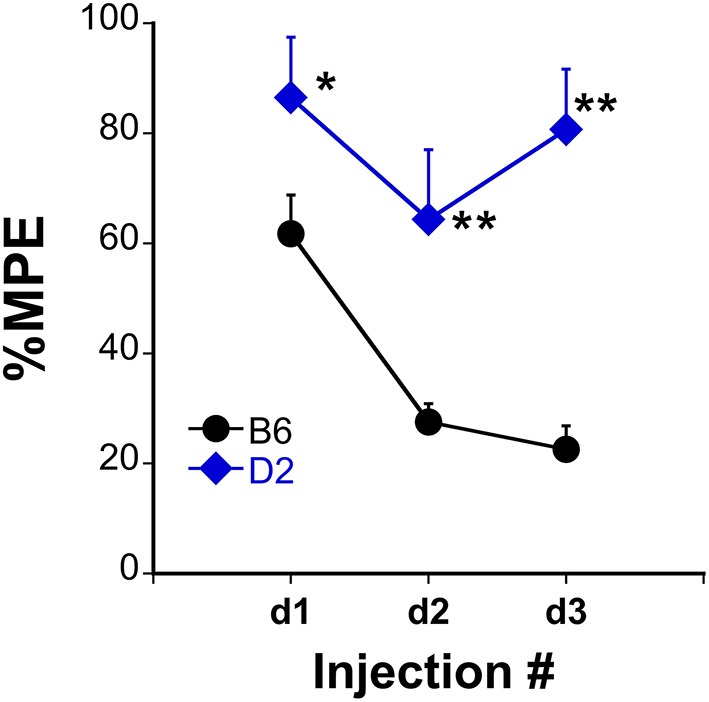
**Tolerance to the analgesic effects of morphine in B6 and D2 mice**. The %MPE following morphine (s.c.) across three injections each spaced 48 h apart significantly decreased in B6 but not D2 treated mice. ^*^P < 0.05, ^**^P < 0.01, significantly different than B6 mice (*n* = 10, 8 for B6, and D2 mice, respectively).

**Figure 3:**
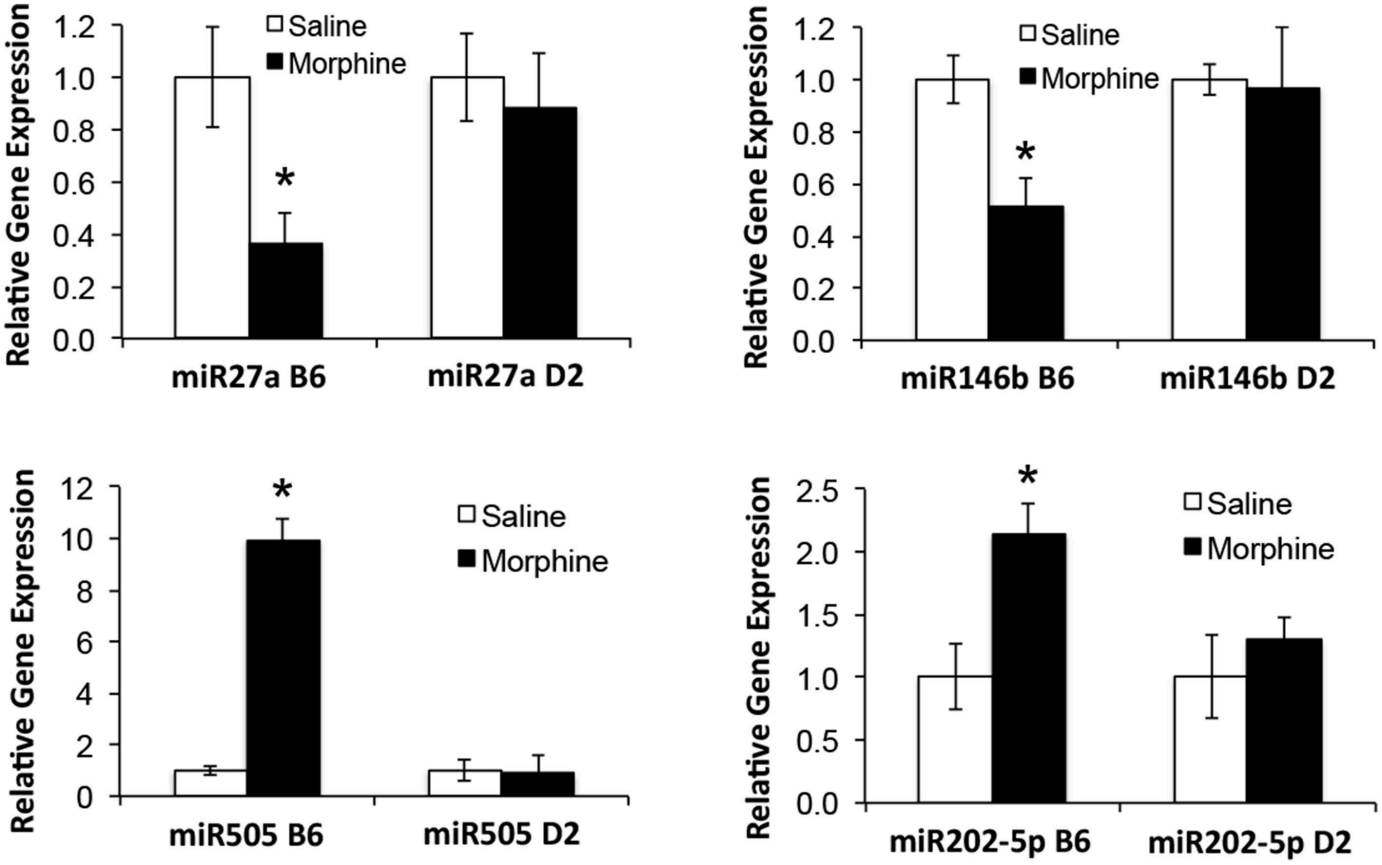
**qRT-PCR validation of miRNAs in prefrontal cortex of B6 and D2 animals treated with chronic saline or chronic morphine**. Data are presented as the mean ± SEM of 4–6 independent animals. ^*^Significantly different from saline treated animals using a Student *t* test, *P* < 0.05. This cohort of animals analyzed by qRT-PCR is an independent, replicate sample from the animals interrogated by miRNA microarray analysis.

Analgesic testing following repeated morphine administration was conducted in B6 and D2 mice to provide brain tissue for miRNA expression profiling and qRT-PCR confirmation of candidate miRNA targets. As previously demonstrated (Tapocik et al., 2009), the analgesic effect of morphine significantly decreased following repeated morphine administration in B6 mice and not in D2 mice [Figure 2; *F*_(*Strain*)_ *df*_(1, 15)_ = 22.02; *p* = 0.0003; *F*_(Injection as repeated measure in B6 mice)_ *df*_(2, 8)_ = 14.91; *p* = 0.002; D2 with injection as repeated measure ns]. To test the hypothesis that miRNAs participate in the behavioral response to COA, miRNA profiling was conducted in a subset of the B6 vehicle and morphine-treated mice (*n* = 4 each). Confirmation of candidate miRNA was conducted in separate B6 mice and contrasted to the level of expression in a genotype that does not develop analgesic tolerance (D2). The miRNAs profiled on Agilent SurePrint arrays, which identified 47 significant differentially expressed miRNAs in the PFC of morphine- vs. saline-treated mice (ANOVA with 10% FDR; 33 up, 14 down) (Table 1). We selected 6 differentially expressed miRNAs for qRT-PCR validation in separate saline and morphine-treated B6 mice (Figure 3). Four out of the six miRNAs undergoing validation exhibited congruence between miRNA profiling and qRT-PCR results, ranging from ~2-fold down-regulation to ~2-fold up-regulation [Table 1 and Figure 3; miR27a: *t*_(10)_ = 2.848, *p* = 0.0173; miR146b: *t*_(10)_ = 3.448, *p* = 0.0063; miR505: *t*_(10)_ = 10.471, *p* = 0.0001; miR202-5p: *t*_(10)_ = 3.222, *p* = 0.0091]. Alterations in miR-126-5p and miR-335-3p were not confirmed. Given that the miRNA profiling and qRT-PCR results were obtained from separate animals, the four out of six validation success rate (67%) can be viewed as reasonable. Furthermore, we took advantage of the qualitative contrast in the development of tolerance between B6 and D2 mice (D2 mice do not develop tolerance under the present conditions, see Figure 2) by quantifying expression of these same miRNAs in D2 animals. COA does not alter miRNA in these non-tolerant D2 providing increased support to these particular miRNA as high value candidates (miR27a; miR146b; miR505; miR202-5p, all ns). Supplementary Figure 1 shows qRT-PCR quantification of miRNAs in PFC of B6 and D2 animals treated with saline. At baseline (saline treated) expression level of miR27a and miR146b were significantly greater in D2 mice compared to the B6 mice while miR505 was equivalent and miR202-5p was significantly less than the B6 mice [miR27a: *t*_(8)_ = 8.411, *p* = 0.0001; miR146b: *t*_(8)_ = 3.448, *p* = 0.0007; miR505: *t*_(8)_ = 0.261, ns; miR202-5p: *t*_(8)_ = 3.000, *p* = 0.0171].

**Table 1:** **miRNA altered by chronic morphine in B6 mice (highlighted miRNA further confirmed by qRT-PCR)**.

**Up-regulated**	**Fold**	**Down-regulated**	**Fold**
mghv miR-M1-9	4.38	miR-382^*^	–1.81
miR-202-5p	4.07	miR-154^*^	−1.49
miR-194	3.46	miR-29b	−0.69
miR-29b^*^	2.44	miR-19b	−0.68
miR-672	2.21	miR-338-3p	−0.59
miR-197	2.13	miR-199b	−0.57
miR-467a-1^*^	1.94	miR-1	−0.52
miR-30b^*^	1.83	miR-34c	−0.48
miR-126-5p	1.76	miR-146a	−0.47
miR-152	1.62	miR-218	−0.40
miR-469	1.55	miR-27a	−0.37
miR-184	1.46	miR-9	−0.37
miR-378^*^	1.37	miR-872	−0.35
miR-669a	1.30	miR-146b	−0.34
miR-92b	1.26		
let-7d^*^	1.26		
miR-505	1.12		
miR-101b	1.05		
miR-429	0.95		
miR-490	0.74		
miR-320	0.73		
miR-22^*^	0.72		
miR-574-3p	0.68		
miR-342-5p	0.67		
miR-376a	0.60		
miR-483^*^	0.58		
miR-700	0.55		
miR-298	0.54		
miR-690	0.53		
miR-380-3p	0.46		
miR-376b	0.43		
miR-423-5p	0.41		
miR-335-3p	0.38		

### Integrative analysis of miRNA and mRNA microarray data

To detect coordinated patterns of miRNA and mRNA expression changes induced by chronic morphine administration, we performed miRNA-mRNA expression pairing analysis with our previous mRNA data set (Tapocik et al., 2009). We identified cases where the expression of a miRNA species and its putative target mRNAs (target prediction based on “Good score” values from microrna.org) were altered in the opposite or same direction following COA. We have previously used this computational approach to identify predicted miRNA-mRNA pairings that had a high probability of successful validation (Wang et al., 2015). We found 30 miRNAs targeting 59 out of 77 mRNAs of our original mRNA data set that fulfilled this condition. The results of the expression pairing analysis are given in Tables 2A,B. Hypergeometric testing determined that seven differentially expressed miRNAs were predicted to “over-target” mRNAs (Supplementary Table 2) as determined by a hypergeometric significant *p*-value (*p* < 0.05), however none of these passed the threshold for FDR.

**Table 2A:** **Negatively correlated miRNA-mRNA pairings in PCR validated miRNAs**.

**miR name**	**miR change**	**miR Log_2_ value**	**mRNA**	**miRSVR score**	**mRNA change**
mmu-miR-146b	↓	−0.3362	Enpp5	−1.0510	↑
	↓		Pet112l	−1.0474	↑
	↓		Afmid	−1.0327	↑
	↓		Ccna2	−0.6094	↑
	↓		Baiap2l1	−0.3049	↑
	↓		Phf17	−0.2832	↑
	↓		Pcbp2	−0.1396	↑
	↓		Dicer1	−0.1357	↑
	↓		C86695	−0.1061	↑
mmu-miR-202-5p	↑	4.0723	Dlgap1	−1.2131	↓
	↑		AI593442	−1.0566	↓
	↑		Rps6ka5	−0.9715	↓
	↑		Meis1	−0.7075	↓
	↑		Fstl1	−0.6275	↓
	↑		Atp2b2	−0.4273	↓
	↑		Rab6b	−0.2834	↓
	↑		Ubqln1	−0.1476	↓
	↑		Ppp1r9a	−0.1379	↓
	↑		Ralgps1	−0.1185	↓
mmu-miR-27a	↓	−0.3690	Fmn2	−0.7565	↑
	↓		Dicer1	−0.7044	↑
	↓		Rufy3	−0.6805	↑
	↓		Dusp9	−0.3455	↑
	↓		Baiap2l1	−0.2262	↑
mmu-miR-505	↑	1.1166	Meis1	−1.2283	↓
	↑		Serpini1	−0.8085	↓
	↑		Ralgps1	−0.4115	↓
	↑		Canx	−0.3364	↓
	↑		Hic2	−0.2643	↓
	↑		Kcnj4	−0.1538	↓
	↑		Vps26a	−0.1231	↓

*Green up arrow: up-regulated in morphine vs. saline. Red down arrow: down-regulated in morphine vs. saline*.

**Table 2B:** **Positively correlated miRNA-mRNA pairings in PCR validated miRNAs**.

**miR name**	**miR change**	**miR Log_2_ value**	**Gene**	**miRSVR score**	**mRNA change**
mmu-miR-146b	↓	−0.3362	Gripap1	−0.6473	↓
	↓		Fstl1	−0.4896	↓
	↓		Dlgap1	−0.4727	↓
	↓		Vps26a	−0.2555	↓
	↓		Gosr2	−0.1763	↓
	↓		Gprasp1	−0.1232	↓
	↓		Zfand6	−0.1105	↓
	↓		Serpini1	−0.1097	↓
mmu-miR-27a	↓	−0.3670	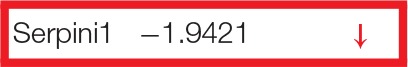		
	↓		Ppp1r9a	−0.8020	↓
	↓		Ubqln1	−0.7975	↓
	↓		Rps6ka5	−0.7940	↓
	↓		Gosr2	−0.7700	↓
	↓		Canx	−0.7310	↓
	↓		AI593442	−0.5274	↓
	↓		Nsf	−0.4902	↓
	↓		Dgkb	−0.4384	↓
	↓		Dlgap1	−0.3411	↓
	↓		Atp2b2	−0.2743	↓
	↓		Zfand6	−0.1456	↓
	↓		Nufip1	−0.1278	↓
mmu-miR-505	↑	1.1166	Sap25	−1.2974	↑
	↑		Parp11	−1.0018	↑
	↑		Srbd1	−0.6136	↑
	↑		Dicer1	−0.3529	↑
	↑		Txnip	−0.2797	↑
	↑		Phf17	−0.1855	↑
	↑		Ccnf	−0.1480	↑

*Green up arrow: up-regulated in morphine vs. saline. Red down arrow: down-regulated in morphine vs. saline*.

**Figure 4:**
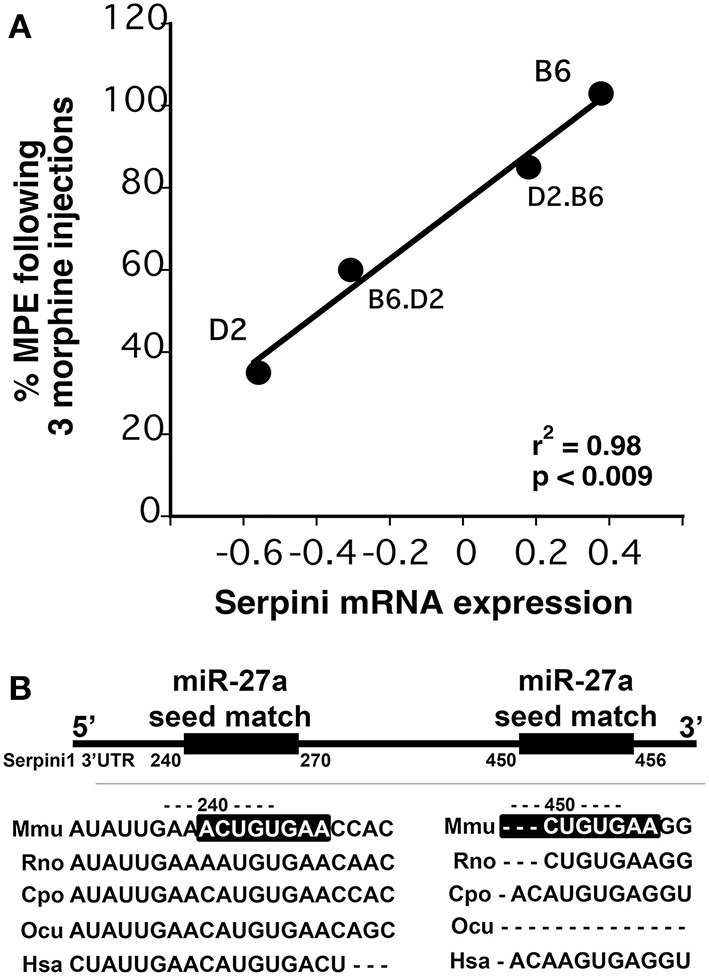
**(A)** Correlation between *Dicer1* mRNA expression and tolerance (as measured by a decrease in the maximal possible effect; %MPE) following 3 morphine administrations in 4 genotypes (data derived from (Tapocik et al., 2009)). **(B)** Map of the putative binding sites of miR-27a on the 3′UTR of Serpini1 and mirSVR scores (derived from microRNA.org). The combined mirSVR score = −1.9421. Mmu, Mus musculus; Rno, Rattus norvegicus; Cpo, Cavia porcellus; Ocu, Oryctolagus cuniculus; Hsa, Homo sapiens.

We chose to investigate and validate the predicted miR-27a-*Serpini1* mRNA pairing due to a number of compelling reasons. First, as regards miR-27a, there were an unusually high number of PFC mRNAs predicted to be targeted by miR-27a (18 targets total). Second, as regards *Serpini1* mRNA, the highest mirSVR score for any target was for *Serpini1*. This particular miRNA was also found to be significantly correlated with analgesic tolerance as determined from data collected in (Tapocik et al., 2009) (Figure 4A). Third, the predicted target, *Serpini1* is contained within a relevant analgesia QTL (Belknap and Crabbe, 1992), and lastly, *Serpini1* is intricately involved in modulating an important neuroplastic element in response to chronic drug administration, namely altered dendritic spine density (see Discussion).

### miR-27a modulates *Serpini1* mRNA expression and protein levels

**Figure 5:**
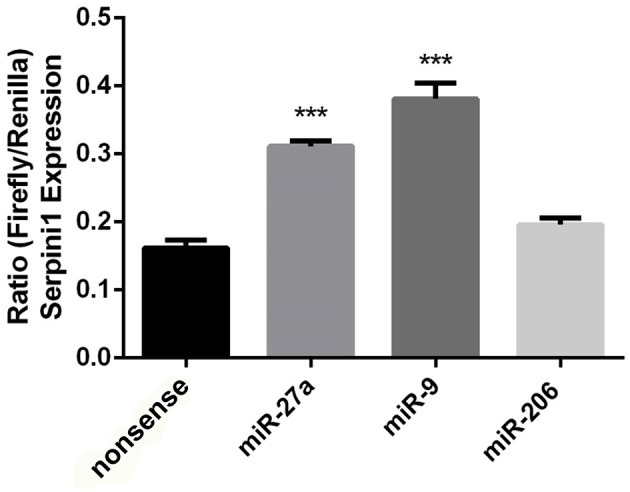
**Putative targeting miRNA's miR-27a and miR-9 increase ***Serpini1*** expression**. Bar plots represent luciferase activity measured in N2A cells cotransfected with either miR-27a, miR-9, miR-206 mimic, or nonsense mimic, and a luciferase reporter plasmid carrying the 3′-UTR of *Serpini1*. *Renilla* luciferase activity was normalized by *firefly* luciferase expression levels and is presented as percentage of activity achieved by the 3′-UTR of *Serpini1* in the presence of mimics (miR27a, miR-9, miR-206, nonsense). MiR-27a and miR-9 target Serpini1 (mirSVR scores −1.9, −0.7, respectively) while miR-206 does not. MiR-27a and miR-9 increased *Serpini1* expression, ^***^p < 0.05.

The mouse *Serpini1* 3′-untranslated region (3′-UTR) is comprised of 1599 nucleotides. Bioinformatics analysis using microRNA.org identified 2 conserved mmu-miR-27a target sites in the Serpini1 3′-UTR, at positions 225 and 435 (Figure 4B). Unexpectedly in a dual luciferase assay employing an mRNA reporter construct of the luciferase open reading frame fused to the *Serpini1* 3′UTR, luciferase protein expression was significantly increased by miR-27a [*t*_(4)_ = 8.46, *p* = 0.011] (Figure 5) and another putative targeting miRNA, miR-9 [*t*_(4)_ = 17.56, *p* < 0.001]; but not miR-206 (*p* > 0.05), a miRNA with no binding sites to *Serpini1*, and a nonsense sequence control.

**Figure 6:**
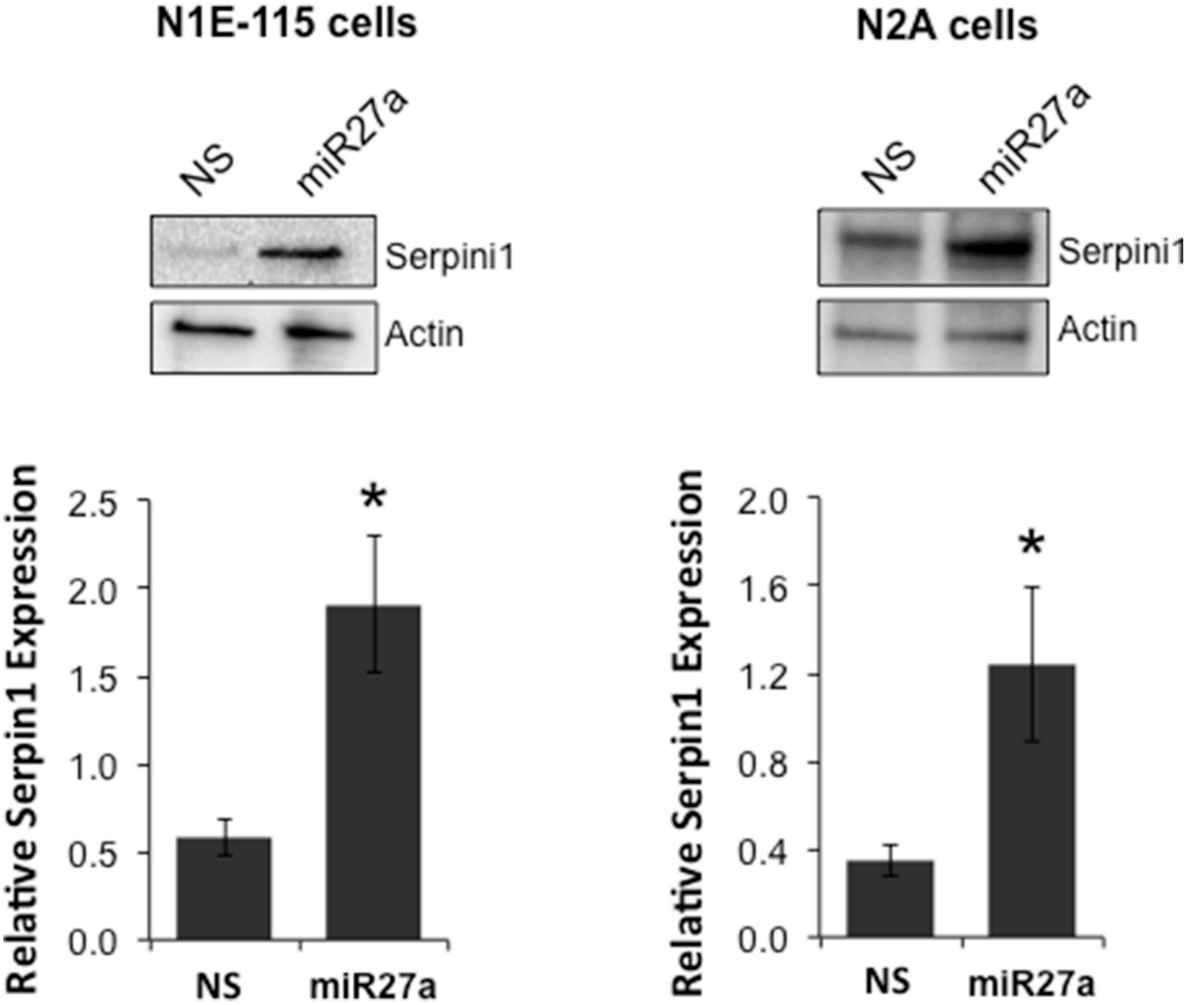
**MiR27a up-regulates Serpini1 protein levels**. Transfection of miR-27a mimic in mouse N1E-115 and N2A neuroblastoma cell lines resulted in up-regulation of Serpini1 protein. **Top** panels, representative western blots from 4 to 8 independent experiments. **Bottom** panels, quantification of relative Serpini1 protein levels was calculated from Serpini1-to-actin densitometric ratios. Data are presented as the mean ± SEM from 4 to 8 independent experiments in N1E-115 and N2A cells, respectively. Each independent experiment is defined as a culture of cells treated with nonsense (NS) miRNA and a separate culture treated with the miR-27a mimic. ^*^Significantly different from nonsense (NS) mimic-treated cell lines using a Student *t* test, *P* < 0.05.

To determine if miR-27a has the ability to increase Serpini1 protein levels, we transfected two cell lines, mouse N2A and N1E-115 neuroblastoma cells, with a miR-27a mimic and measured protein levels of Serpini1. MiR-27a significantly increased the level of Serpini1 protein by >3-fold compared to cells transfected with nonsense mimic control [N1E-115: *t*_(6)_ = 3.26, *p* = 0.017, N2A: *t*_(14)_ = 2.40, *p* = 0.031, Figure 6].

**Figure 7:**
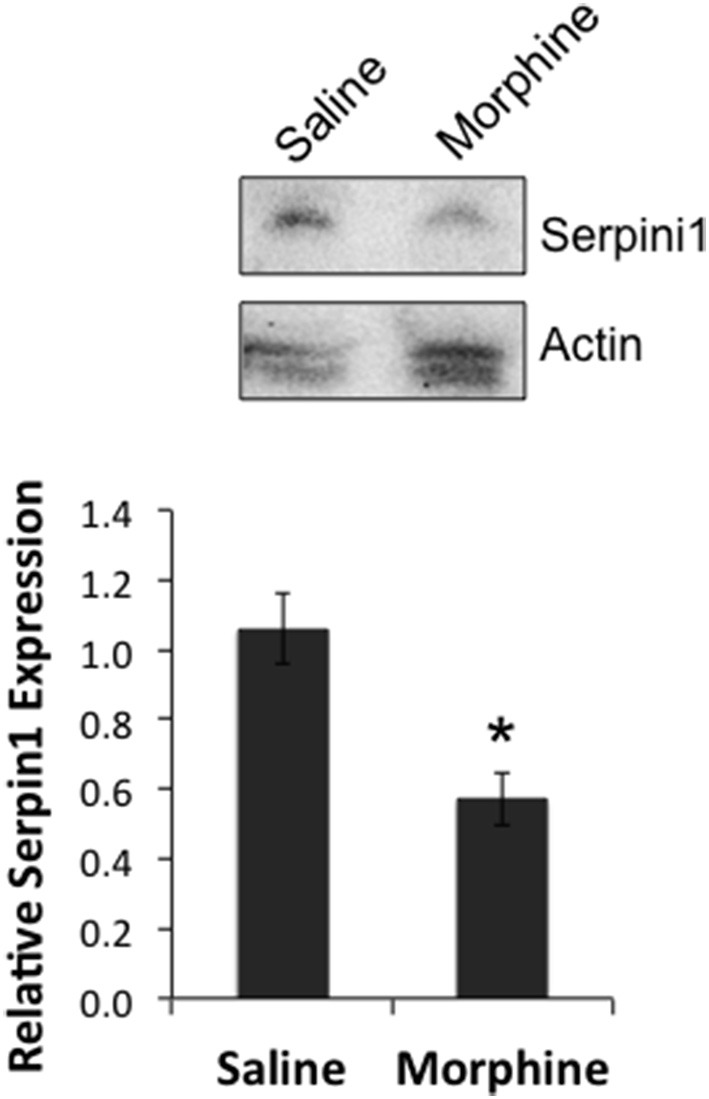
**Chronic morphine down-regulates Serpini1 in prefrontal cortex of B6 animals. Top** panel, representative western blot from 4 to 8 independent animals. **Bottom** panel, quantification of relative Serpini1 protein levels was calculated from Serpini1-to-actin densitometric ratios. Data are presented as the mean ± SEM from 4 saline-treated and 4 morphine-treated animals. ^*^Significantly different from saline-treated animals using a Student *t* test, *P* < 0.05.

In order to determine if there was a link between the proposed chain of events (COA → decreased miR-27a → decreased Serpini1 mRNA → decreased Serpini1 protein), Serpini1 protein levels were characterized in analgesic tolerant B6 mice [analgesic tolerance: *n* = 6 saline, 7 morphine; *F*_(Injection #)_ *df*_(2, 6)_ = 4.965; *p* = 0.0047; data not shown, results not different from those shown in Figure 2]. From these phenotyped animals, we harvested high quality PFC from *n* = 4 saline-treated and *n* = 4 morphine-treated animals for western analysis of Serpini1 protein levels. COA resulted in a significant decrease in PFC Serpini1 protein compared to saline-treated animals [*t*_(6)_ = 3.36, *p* = 0.015, Figure 7].

**Figure 8:**
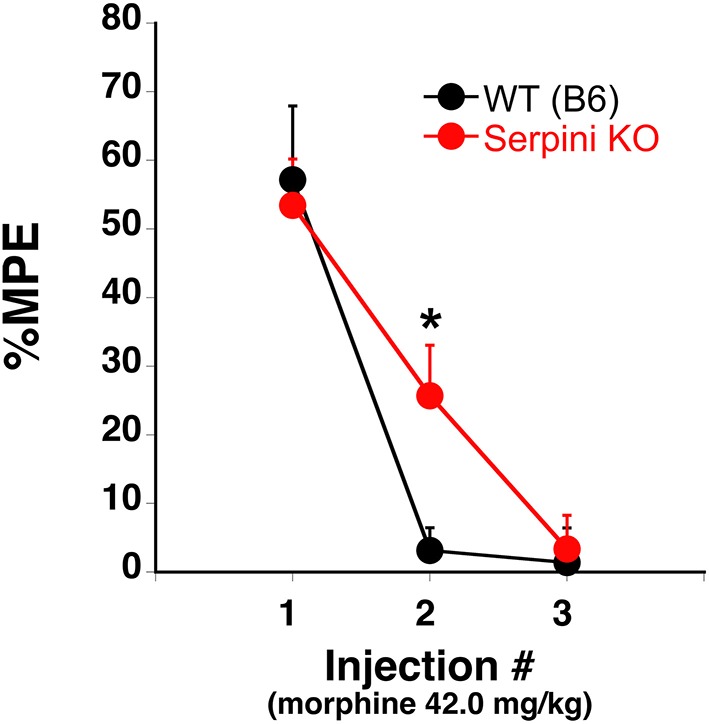
**Tolerance to the analgesic effects of morphine in B6 (WT) and Serpini KO mice**. When the 42.0 mg/kg (s.c.) morphine is given once every other day for a total of 3 injections B6 develop tolerance [decrease in the % maximal possible effect (%MPE)] at a faster rate than Serpini KO mice (mean ± SE; *n* = 8 and 6, KO and B6, respectively). ^*^Significantly different from WT mice, *P* < 0.05.

Our findings can be summarized as follows: (a) COA decreases the expression of miR-27a in mice that develop tolerance (Figures 2, 3), (b) miR-27a increases, not decreases, serpini1 expression (Figures 5, 6), thus decreased miR-27a may decrease Serpini1 expression, (c) COA decreases Serpini1 protein levels in the PFC (Figure 7), and (d) Serpini1 is negatively correlated with tolerance (less Serpini1 is associated with greater tolerance; Figure 4A). Thus, a decrease in miR-27a may lead to a decrease in Serpini1 that in turn leads to tolerance. In order to determine if there was a causal association between miR-27a alteration of Serpini1 expression and the development of analgesic tolerance, we characterized the development of tolerance in Serpini1 KO animals and the corresponding B6 control group (Figure 8). Baseline nociception did not differ between the genotypes. Acute morphine administration (42.0 mg/kg) resulted in equivalent degree of analgesia following the first injection, thus Serpini1 knockout did not alter acute sensitivity. In contrast, chronic morphine administration (42.0 mg/kg per injection) led to a rapid and full degree of tolerance by the second injection in the B6 mice, however Serpini1 KO mice took three injections to reach the same degree of full tolerance [ANOVA *F*_(Genotype × Injection #)_ *df*_(2, 10)_ = 4.078; *p* = 0.050; *post-hoc* Injection # 2 KO vs. B6 *p* = 0.0387]. These results suggest that the process of reducing Serpini1 is a necessary factor in the development of tolerance.

## Discussion

The overall goal of the study was to determine if miRNAs are involved in mediating the neurobiological mechanisms responsible for the development of analgesic tolerance following COA. Two key findings support their involvement; first, decreasing the expression of the rate-limiting enzyme in miRNA production, Dicer1, prevents the development of tolerance and second, the expression of a select number of miRNAs is altered following the administration of pharmacologically-relevant doses of morphine. Further confirmation was obtained by demonstrating differential expression of key miRNAs in tolerance susceptible (B6) vs. unsusceptible genotypes (D2). Interestingly, despite COA producing an increase in *Dicer1* in tolerant mice, 14 of the 47 miRNAs significantly altered by COA were down-regulated. The discord between *Dicer1* levels and down-regulated miRNAs could reflect Dicer-independent processing (Bhinder et al., 2014; Liu et al., 2015) that may even give rise to inverse production levels (possibly due to competitive Dicer loading; Liu et al., 2015). This point is relevant since we chose to further investigate a miRNA that was down-regulated, namely miR-27a. Several factors led us to investigate miR-27a, such as the very high number of putative mRNA targets within previously identified COA-relevant mRNAs in the same brain region and its putative targets involved in neuroplasticity. Furthermore, we chose to investigate its predicted mRNA target, *Serpini1*, due to its high mirSVR score and role in neuroplasticity (Parmar et al., 2002; Navarro-Yubero et al., 2004; Borges et al., 2010; Lee et al., 2012). The experiments confirmed miR-27a regulation of *Serpini1* mRNA and protein as well as demonstrating altered analgesic tolerance development in Serpini1 KO animals. Overall, the study supports a significant and targetable role for miRNA in the development of analgesic tolerance.

The neurobiological consequences of COA likely involve coordinated and complex neuroadaptations. Altered gene expression is a plausible means for inducing these long-term changes. Studies conducted by our laboratory (Tapocik et al., 2009, 2013) revealed a network of gene expression changes occurring in canonical pathways involved in neuroplasticity, and uncovered miRNA processing as a prime candidate coordinating these changes in response to COA. In particular, mRNA coding the protein responsible for processing miRNAs, *Dicer1*, was positively correlated with the development of analgesic tolerance in the PFC. These results along with several studies demonstrating miRNA involvement in cellular response to acute opioid administration (Wu et al., 2008, 2009, 2013; Sanchez-Simon et al., 2010; Hwang et al., 2012; Zheng et al., 2012; Ni et al., 2013; Gonzalez-Nunez et al., 2014; Lu et al., 2014; Xu et al., 2015) and miRNA involvement in neuroplasticity that may be relevant to decreased spine density seen in the PFC (Robinson et al., 2002; Robinson and Kolb, 2004), gave rise to the hypothesis that key miRNAs modulate the development of analgesic tolerance. As a first test of this hypothesis, regionally targeted *Dicer1* knockdown (via shRNA) had the anticipated consequence of eliminating the development of tolerance in B6 mice. These *in vivo* findings strongly supported the involvement of miRNA processing in the acquisition of tolerance. MiRNA expression profiling following therapeutically relevant doses identified a small, core set of COA-regulated miRNAs (~6% of the characterized miRNAs). Bioinformatic tools were utilized to identify miRNAs with a high number of valued mRNA targets in our previous microarray study for qRT-PCR validation (miR's 27a, 9, 483, 505, 146b, 202). Further corroboration of their importance in analgesic tolerance was confirmed by the lack of expression change in a genotype that does not develop tolerance following pharmacologically-relevant doses (D2; Tapocik et al., 2009).

Several miRNAs have previously been demonstrated to moderate the consequences of COA (Hwang et al., 2012). For example, COA-induced increases in a miRNA postulated to alter OPRM1 mRNA expression, Let-7, correlates with analgesic tolerance and targeted deletion of Let-7 attenuates the development of analgesic tolerance (He et al., 2010; He and Wang, 2012). In the current study Let-7b was increased and two additional putative OPRM1 mRNA targeting miRNAs were decreased [miR-154 and miR-199b, (He and Wang, 2012)] but not miR-134, (Ni et al., 2013). Additional studies demonstrate altered miRNA expression in CNS tissue following opioid administration; miR-190 (Zheng et al., 2010a), miR-133b (Sanchez-Simon et al., 2010), miR-28, -125b, -150 and -382 (Wang et al., 2011), miR-23b and -339 (Wu et al., 2008, 2009, 2013), miR-339 (Zheng et al., 2012), miR-103 and -107 (Lu et al., 2014), miR-21 and -146a (Strickland et al., 2014), and miR-124 (Qiu et al., 2015). However, none of the aforementioned miRNAs were found to be differentially-regulated in the current study. Treatment conditions and tissue source are likely explanations for the discrepancies, for example miR-103 and -107 were only differentially expressed in striatum and not PFC (Lu et al., 2014). MiRNAs from macrophages were altered by morphine (miR-146a and -423-5p; Dave and Khalili, 2010) and in our study as well (although in opposite directions). The current set of experiments are the first to report the confirmed association of miRNAs miR-27a, -9, -483, -505, -146b, and -202) using pharmacologically-relevant doses of morphine.

Once a core set of miRNAs were identified and confirmed we performed miRNA-mRNA expression pairing analysis with our previously collected mRNA data to identify cases where the expression of a miRNA species and its putative target mRNAs (derived from microrna.org) were altered in the opposite or same direction following COA (Wang et al., 2015). We chose to investigate and validate the predicted miR-27a-Serpini1 mRNA pairing due to (a) the unusually high number of PFC tolerance-related mRNAs predicted to be targeted by miR-27a (18 out of 78), (b) the high miRSVR score for miR-27a targeting Serpini1, (c) the fact that the Serpini1 is contained within a relevant analgesia QTL (Belknap and Crabbe, 1992; Belknap et al., 1995; Bergeson et al., 2001), (d) the demonstrated positive correlation between Serpini1 mRNA levels and the prevention of analgesic tolerance (see Figure 4A), and (e) the fact that Serpini1 is intricately involved in modulating an important neuroplastic element in response to chronic morphine administration, namely altered dendritic spine density (Robinson et al., 2002; Robinson and Kolb, 2004; Ballesteros-Yanez et al., 2007; Zheng et al., 2010b; Kobrin et al., 2015). *In vitro* reporter assay confirmed the targeting of the *Serpini1* 3′-untranslated region by miR27a. Interestingly, miR27a was found to *positively* regulate *Serpini1* mRNA and protein levels in multiple neuronal cell lines and to be associated with the development of morphine-induced analgesic tolerance.

The up-regulation of *Serpini1* mRNA and protein by miR27a may appear at first sight surprising, given that miRNAs have typically been associated with down-regulation of gene expression (i.e., translation repression and/or mRNA degradation) (He and Hannon, 2004). More recently, notable examples of translational activation by miRNAs have appeared in the literature (Vasudevan et al., 2007; Orom et al., 2008; Jacobsen et al., 2010). These miRNAs have been shown to bind *cis*-acting sites that are adjacent/overlapping AU-rich motifs in the 3′-UTR of the targeted mRNA, as is the case for miR27a/*Serpini1* 3′-UTR (see Figure 4B). AU-rich motifs near miRNA binding sites enhance translational efficiency of the targeted mRNA by mechanisms that remain to be fully elucidated (Vasudevan et al., 2007; Jacobsen et al., 2010).

The results thus far support the hypothesis that in mice readily developing tolerance under pharmacologically-relevant conditions, COA decreases the expression of miR-27a, which in turn decreases *serpini1* mRNA and protein expression thereby leading to analgesic tolerance. In order to determine if there was a causal association between miR-27a alteration of Serpini1 expression and the development of analgesic tolerance, we characterized the development of tolerance in Serpini1 KO animals. Several possible outcomes were hypothesized to occur; (a) lack of analgesic effect in KO mice upon first injection that would reflect an already “tolerant” state, (b) a faster rate of tolerance if Serpini1 needs to be at a certain level to show tolerance or (c) a slower rate of tolerance since the dynamic process of reducing or withdrawing Serpini1 is a necessary factor in the development of tolerance or (d) no effect due to developmental compensation. Acute morphine administration resulted in equivalent degree of analgesia, thus *Serpini1* knockout did not alter acute sensitivity (eliminate “a” as possibility). In contrast, chronic morphine administration led to a rapid and full degree of tolerance by the second injection in the B6 mice, whereas Serpini1 KO mice took three injections to reach the same degree of full tolerance (eliminating “b,” supporting “c”). These results provide evidence to support a role for miR27a and *Serpini1* in the behavioral response to COA.

MiRNAs are prime candidates to mediate network-like changes in response to chronic drug administration. These molecules are thought to regulate as much as one-third of the transcribed genome, can mediate short and long term neuroplasticity and play a role in sculpting the dendritic architecture (Kosik, 2006; Impey et al., 2010; Im and Kenny, 2012; Weiss et al., 2015; Xu et al., 2015). Differential miRNA expression/processing and mRNA targeting may underlie the neuroadaptations that mediate altered dendritic morphology in the PFC and tolerance to the analgesic effects of morphine. MiR-27a induced alterations in Serpini1 (also referred to as neuroserpin) may alter neural signaling as its overexpression significantly influences the levels of a major postsynaptic scaffolding protein, postsynaptic density protein 95 (Tsang et al., 2014), and affects the density and shape of dendritic spines (Borges et al., 2010) and neurite outgrowth (Parmar et al., 2002; Navarro-Yubero et al., 2004; Lee et al., 2012). An increase in Serpini1 has been shown to increase the density of dendritic protusions (Borges et al., 2010). Accordingly, our findings that COA decreased both miR-27a and Serpini1 protein aligns with previously observed decreases in PFC cortex spine density following COA (Robinson et al., 2002). Altered synaptic profile in the PFC may influence the cognitive/associated role that plays a factor in the development of tolerance (Tiffany et al., 1991; Cox and Tiffany, 1997; Mitchell et al., 2000; Miguez et al., 2014). Interestingly, a lower baseline level of miR-27a was observed in B6 vs. D2 animals (Supplementary Figure 1), which may explain the lower sensitivity of B6 to the analgesic effects of morphine (i.e., B6 ED90 is 31.2 mg/kg while D2 ED90 is 7.7 mg/kg).

Similar neuroadaptive roles can be inferred from the miRNAs confirmed by qRT-PCR. In particular, miR-483 presents an interesting link between our mRNA findings and current miRNA results. MiR-483 resides in the second intron of imprinted *Igf2* and is known to be a regulatory factor for *Igf2/H19* expression (Veronese et al., 2010; Ma et al., 2011; miR-483 overexpression increases *Igf2*). In our previous study, high H19 expression was predictive of tolerance development and H19 expression decreased in the genotypes that became tolerant to morphine and increased in those that did not. Thus, an increase in miR-483 (as observed in present study) would be expected to down-regulate *Igf2*/H19 expression consistent with the direction seen in the development of tolerance.

MiR-505, -146b, and -202 are proposed to be involved in various adaptive processes such as angiogenesis (miR-505; Yang et al., 2014), dendritic cell apoptosis and cytokine production (146b; Park et al., 2015) and cell proliferation (Zhang et al., 2014). MiR-9 was found to be overexpressed following COA and specifically by miR-27a (Figure 5). MiR-9 is highly expressed in the nervous system and plays essential roles in neurogenesis, axon growth, dendritic development and regulation of the gene silencing transcription factor REST [repressor element 1 silencing transcription factor]/NRSF (neuron-restrictive silencer factor) that represses a large array of coding and noncoding neuron-specific genes important to synaptic plasticity (Giusti et al., 2014; Wang et al., 2016). MiR-9 has also been implicated in regulation of the dynorphin κ-opioid receptor system via down-regulation of REST (Henriksson et al., 2014). Increased miR-9 would down-regulate REST and result in a net increase in prodynorphin expression, a result consistent with some morphine dosing patterns in a region specific manner (Przewłocka et al., 1996; Király et al., 2006). Overall, it will be important for future studies to consider the interplay between various candidate miRNA and characterize their time-course as it relates to the development of morphine tolerance.

Overall, our results demonstrate for the first time the *in vivo* role of *Dicer1* in the development of tolerance. Furthermore, we identified a significant alteration in miRNA expression that may play a significant regulatory role in the development of analgesic tolerance to morphine. The miRNA expression data generated in this study can serve as a future resource to elucidate mechanisms involved in morphine tolerance. In particular, a specific role for miR27a and *Serpini1* in the behavioral response to COA is proposed and suggest that a larger role for differential miRNA expression and mRNA targeting may underlie the neuroadaptations that mediate tolerance to the analgesic effects of morphine.

## Author contributions

JT, NL, and GE were responsible for the study concept and design. JT and CM contributed to the acquisition of animal data. KC, TL, JO, TH, and BW performed the microarray, qRT-PCR experiments, and western studies. JT and MS contributed to the luciferase assays. JT, CM, TH, and TM contributed to histochemical characterization. JT, MLS, NL, and GE were responsible for data analysis and interpretation of findings. JT, NL, and GE drafted the manuscript. JT, NL, and GE provided critical revision of the manuscript for important intellectual content. All authors critically reviewed content and approved final version for publication.

### Conflict of interest statement

The authors declare that the research was conducted in the absence of any commercial or financial relationships that could be construed as a potential conflict of interest.

## Supplementary material

Supplementary Figure 1 **qRT-PCR quantification of miRNAs in prefrontal cortex of B6 and D2 animals treated with saline**. Data are presented as the mean ± SEM of 4–6 independent animals. ^*^Significantly different from saline treated animals using a Student *t*-test, *P* < 0.05. These cohort of animals analyzed by qRT-PCR are the same animals analyzed in Figure 3.

Supplementary Table 1 **Primer sequences for qRT-PCR validation of miRNAs**.

Supplementary Table 2 **Hypergeometric testing to identify differentially expressed miRNAs that ‘over-target’ differentially expressed mRNAs**.
