# Supplementary figures and images for: MicroRNAs Are Involved in the Development of Morphine-Induced Analgesic Tolerance and Regulate Functionally Relevant Changes in Serpini1
Source: Front Mol Neurosci. 2016 Mar 24;9:20. doi: 10.3389/fnmol.2016.00020 (PMC4805586; doi:10.3389/fnmol.2016.00020)

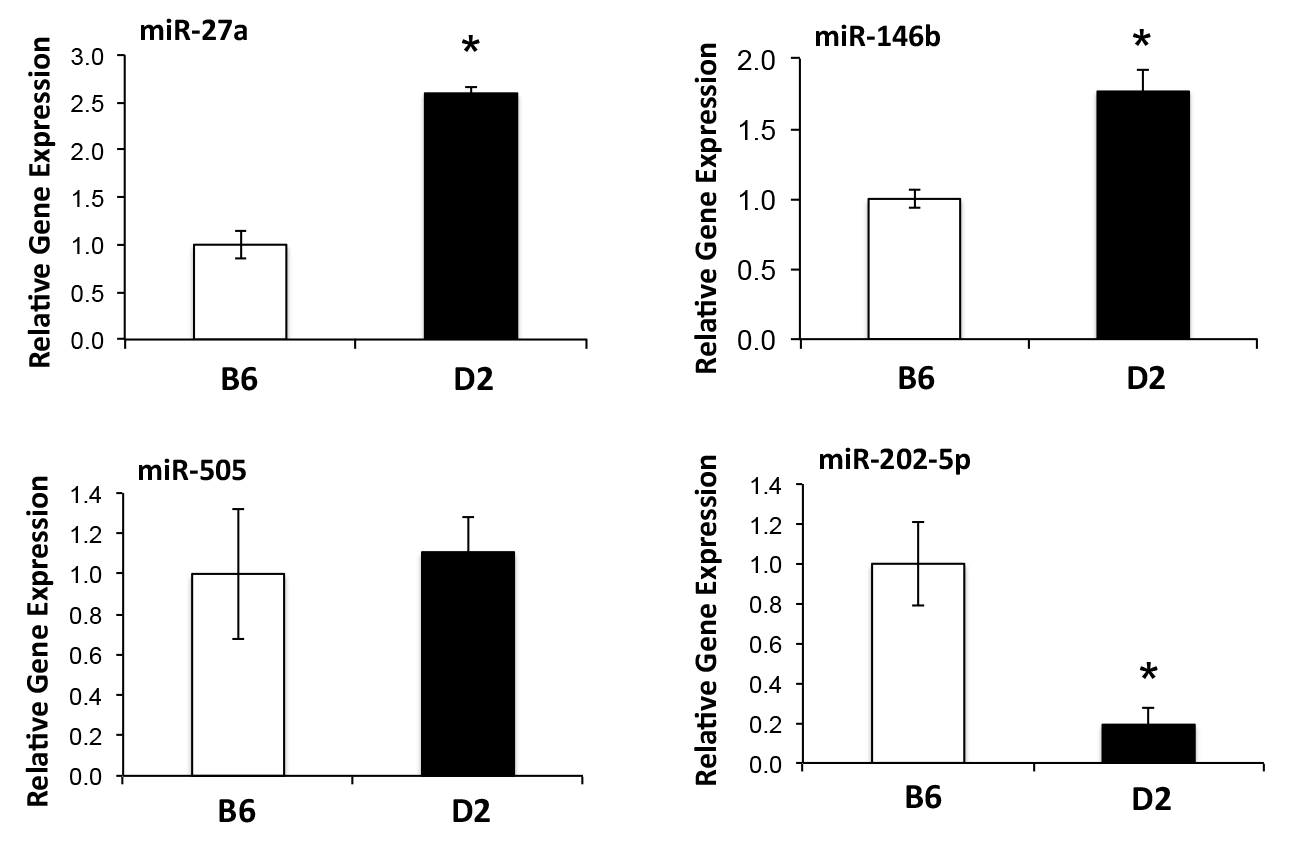

Supplement: Supplementary Figure 1 — qRT-PCR quantification of miRNAs in prefrontal cortex of B6 and D2 animals treated with saline. Data are presented as the mean ± SEM of 4–6 independent animals. *Significantly different from saline treated animals using a Student t-test, P < 0.05. These cohort of animals analyzed by qRT-PCR are the same animals analyzed in Figure 3. [file Image1.TIF]
