# Supplementary material for: MicroRNAs Are Involved in the Development of Morphine-Induced Analgesic Tolerance and Regulate Functionally Relevant Changes in Serpini1
Source: Front Mol Neurosci. 2016 Mar 24;9:20. doi: 10.3389/fnmol.2016.00020 (PMC4805586; doi:10.3389/fnmol.2016.00020)
Supplement: Supplementary Table 1 — Primer sequences for qRT-PCR validation of miRNAs. [file Table1.DOCX]

**Supplementary Table 1. Primer sequences for qRT-PCR validation of miRNAs.**

| **miRNA** | **Primer sequences (forward, 5' to 3')** |
| --- | --- |
| mmu-miR-27a | TTCACAGTGGCTAAGTTCCGC |
| mmu-miR-146b | TGAGAACTGAATTCCATAGGCT |
| mmu-miR-505 | CGTCAACACTTGCTGGTTTTCT |
| mmu-miR-202-5p | TTCCTATGCATATACTTCTTT |
